# Sustained Delivery of Liraglutide Using Multivesicular Liposome Based on Mixed Phospholipids

**DOI:** 10.3390/pharmaceutics17020203

**Published:** 2025-02-06

**Authors:** Runpeng Zhang, Xinyu Yao, Siqi Gao, Tingting Xu, Da Wang, Luping Sha, Li Yang

**Affiliations:** 1 School of Pharmacy, Shenyang Pharmaceutical University, Shenyang 110016, China; rpzhang1115@aliyun.com (R.Z.); xyy78100210@aliyun.com (X.Y.); ella545@aliyun.com (S.G.); xutingting@syphu.edu.cn (T.X.); 101030307@syphu.edu.cn (D.W.); 2 School of Pharmaceutical Engineering, Shenyang Pharmaceutical University, Shenyang 110016, China

**Keywords:** peptides, sustained release, multivesicular liposomes, liraglutide, type 2 diabetes mellitus

## Abstract

**Background:** Although peptides are widely used in the clinical treatment of various diseases due to their strong biological activity, they usually require frequent injections owing to their poor in vivo half-life. Therefore, there is a strong clinical need for sustained peptide formulations. **Methods:** In this study, liraglutide (Lir) and biocompatible multivesicular liposomes (MVLs) were utilized as the model drug and sustained-release carriers, respectively. The drug release rate of Lir-MVLs was controlled by changing the ratio of SPC and DEPC with different phase transition temperatures (PTT, PTT_SPC_ = −20 °C, PTT_DEPC_ = 13 °C). **Results:** As the SPC ratio increased, Lir-MVLs had more flexible lipid membranes, poorer structural stabilization, and fewer internal vesicles with larger particle sizes, contributing to faster release of Lir. After subcutaneous injection of Lir-MVLs, the blood glucose concentration (BGC) of db/db mice decreased to different levels. When the SPC-DEPC ratio was greater than 85:15, the drug release rate was too fast; the BGC remained below 16 mM for only 2–4 days, while when the drug release rate was too slow, was the case when the SPC-DEPC ratio was less than 50:50, the BGC also remained below 16 mM for only 2–3 days. However, when the SPC-DEPC ratio was 75:25, the BGC could be maintained below 16 mM for 8 days, indicating that the release properties of this ratio best met the pharmacological requirements of Lir. **Conclusions:** This study investigated the effects of phospholipids with different PTT on the release characteristics of Lir-MVLs, and provided ideas for the design of sustained-release peptide preparations.

## 1. Introduction

The application of peptides in the treatment of various diseases is gradually gaining attention. For example, Glucagon-like peptide-1 is an endogenous enteric insulin that plays an important role in maintaining glucose stability in the body, and its analogs, such as Liraglutide (Lir) and Semaglutide (Sem), are used in the clinical treatment of type 2 diabetes mellitus [1,2]. Teriparatide and leucovorin have also been approved on the market for the anabolic treatment of osteoporosis and for the treatment of prostate cancer, respectively [3,4]. Peptides are macromolecules with secondary or tertiary structures, which can rapidly and efficiently bind to receptors in vivo, granting them unique target specificity, potent effects, and low toxicity [5,6]. In recent years, delivery systems for peptides such as microspheres based on polylactide-co-glycolide (PLGA) or polylactic acid [7], PEGylation [8], chitosan nanoparticles [9,10], microneedle-mediated transdermal [11], and exosome nanovesicles [12] have been gradually developed to improves bioavailability.

The advanced structure of peptides is the basis for maintaining their pharmacological efficacy, but they are also highly susceptible to disruption by the physiological environment of the body, which contributes to their short biological half-life [13]. Therefore, peptide drugs typically require frequent administration, resulting in poor patient compliance. Thus, the development of sustained-release platforms for peptide drugs has strong market and clinical values. PLGA is currently the most widely used sustained-release peptide delivery platform due to its biocompatibility and biodegradability, but it is possible that the degradation of PLGA may lead to the production of lactic acid and glycolic acid, the excessive accumulation of which can lead to local pH variation and inflammation [14,15]. Therefore, to avoid the above problems, phospholipid-based sustained-release delivery systems for peptides have been gradually developed [16,17]. Phospholipids are biocompatible and safe, and their metabolites do not alter the physicochemical environment at the site of administration, making them highly suitable for the delivery of peptides that require long-term administration [18,19,20]. Moreover, phospholipids spontaneously form bilayers under certain conditions and can be prepared as long-lasting subunit formulations by the double-emulsification method.

**Figure 1:**
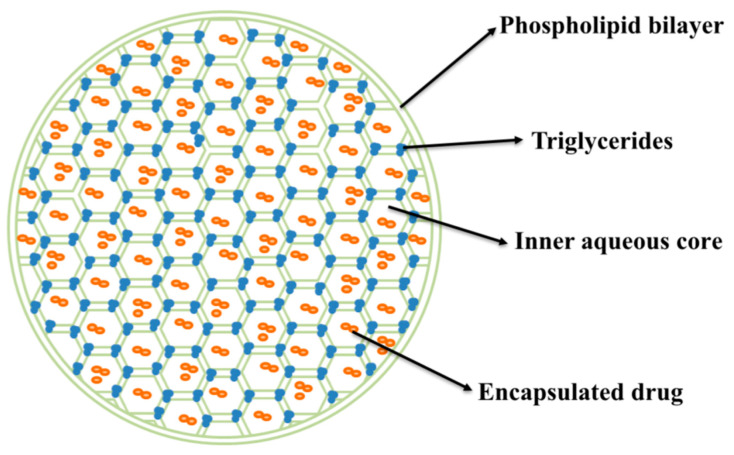
Structure of multivesicular liposomes.

The multivesicular liposomes (MVLs) phospholipids-based sustained-released drug-delivery system prepared by DepoFoam technology is shown in Figure 1 [21]. The sphere is composed of numerous non-concentric aqueous chambers separated by a reticulated lipid membrane, with adjacent chambers sharing a common phospholipid bilayer, and the vesicles are tightly stacked to form a stable topological structure [22]. MVLs are characterized by large aqueous chambers, a smooth slow-release performance, mild preparation conditions, excellent biocompatibility, and pH-neutral metabolites [23,24], making them suitable for encapsulating protein and peptide drugs [25,26]. The rupture of the surrounding lipid membranes leads to the release of the drug only in the peripheral chambers, while the internal chambers are not affected, prolonging the release time of the aqueous chamber; this makes them a good reservoir for hydrophilic drugs, which are highly suitable for long-term treatment of peptides [21,27,28,29,30]. Preparation of MVLs requires the use of amphiphilic phospholipids as the main membrane material that forms lipidic bilayer membranes. In the meantime, anionic-charged phospholipids are used to form metastable monolayers during preparation, and cholesterol provides fluidity and rigidity to the membrane. In contrast to other liposomes, neutral lipids are present at the junction of bilayers within vesicles, forming MVLs, which can maintain the structural stability of MVLs [23].

The in vitro release mechanisms of MVLs generally occur through erosion and diffusion [31,32,33], and current studies on the release characteristics of MVLs have mainly focused on the neutral lipid [34], the inner aqueous phase [35], and particle size [36]. Phospholipids’ structure is diverse, with different phase transition temperatures (PTTs), endowing them with different flexibility and fluidity [37], which influences drug release behavior. Meanwhile, the vesicles formed by phospholipid membrane encapsulation are the basic drug release unit of MVLs; thus, the nature of the phospholipid membrane has a great influence on their drug release. We hypothesized that by using two mixed phospholipids with different PTTs as membrane materials and adjusting the ratio of them, the ability to regulate drug release more finely was achieved, thereby resulting in a MVLs delivery system with a sustained-release effect.

Therefore, in this paper, the effect of adjusting the ratio of mixed phospholipids on the slow-release properties of MVLs was investigated. Specifically, Lir and MVLs were utilized as the model drug and sustained-release carriers, respectively. A series of Lir-MVLs were prepared using different Soybean phospholipids (SPC, PTT = −20 °C) and dierucoyl phosphatidylcholine (DEPC, PTT = 13 °C) ratios by the double-emulsification method. The lipid membrane fluidity, surface properties, and characterization of aqueous phase–particle interfaces of Lir-MVLs based on mixed phospholipids were investigated by adjusting the proportion of SPC-DEPC in the formulation. By combining an in vitro release test and an in vivo hypoglycemic experiment, the relationship between the membrane structural features of Lir-MVLs and their release behavior was investigated in order to meet different clinical needs. In summary, we designed and prepared Lir-MVLs with good hypoglycemic effect and sustained-release performance, opening up a promising strategy for the design of an injectable sustained-release drug-delivery system in peptides.

## 2. Materials and Methods

### 2.1. Materials

Lir (>99%) was kindly gifted by Huadong Pharmaceutical Co., Ltd. (Hangzhou, China). SPC was purchased from Shenyang Tianfeng Bio-Pharm Co., Ltd. (Shenyang, China). DEPC, and 1,2-palmitoyl phosphatidylglycerol (DPPG) were provided by Lipoid Co., Ltd. (Ludwigshafen, Germany). Cholesterol (CHO) was purchased from Shanghai Dongshang Biotechnology Co., Ltd. (Shanghai, China). Triolein (TO) was supplied by Shanghai Ruiyong Biotechnology Co., Ltd. (Shanghai, China). Human serum albumin (HSA), L-lysine, Cyanine 5, and Coumarin 6 were offered by Dalian Meilun Biotechnology Co., Ltd. (Dalian, China). Glucose was purchased from Tianjin Hengxing Chemical Reagent Manufacturing Co., Ltd. (Tianjin, China). Trichloromethane was purchased from Tianjin Lianlong Bohua Pharmaceutical Chemical Co., Ltd. (Tianjin, China).

### 2.2. Preparation of Lir-MVLs

It has been reported that the double-emulsification method is currently used to prepare MVLs encapsulating protein or peptide drugs [25,26,28]. Briefly, 7 mg·mL^−1^ Lir and 18 mg·mL^−1^ HSA were dissolved in phosphate buffer to form the inner aqueous phase, while mixed phospholipids (SPC and DEPC), DPPG, CHO, and TO were dissolved in trichloromethane to form the organic phase. The inner aqueous phase was mixed with the organic phase and dispersed (SCIENTZ-IID ultrasonic cell pulverizer, Ningbo Xinzhi Bio-technology Co., Ltd., Ningbo, China) to obtain the first *w*/*o* emulsion. The first emulsion and the external aqueous phase containing 5% Glucose and 40 mM L-Lysine were stirred to form the second *w*/*o*/*w* emulsion. After the second emulsion was flushed with nitrogen, the external aqueous phase was replaced with saline to obtain the final preparation, which was stored at 4 °C. By keeping the mixed phospholipid concentration unchanged and varying the molar ratio of SPC and DEPC, Lir-MVLs were prepared with pure SPC, pure DEPC, and SPC-DEPC molar ratios of 85:15, 75:25, 50:50, and 25:75 (denoted as SPC 85:15 DEPC, SPC 75:25 DEPC, SPC 50:50 DEPC, and SPC 25:75 DEPC).

### 2.3. Physiochemical Characterization

For multi-unit sustained-release formulations like MVLs, encapsulation efficiency (EE) and the particle size are critical pharmaceutical indicators for evaluating the manufacturing process. EE was calculated according to Equation (1), where M_T_ represents the total drug amount and M_E_ represents the encapsulated drug amount. Lir was detected by HPLC on an C18 column (4.6 × 250 mm, 5 μm) (Agilent, Santa Clara, CA, USA), and the mobile phase was water–acetonitrile (55:45, *v*/*v*, containing 0.1% trifluoroacetic acid).

EE (%) = M_E_/M_T_ × 100 (1)

The particle size was determined by using a laser particle sizer (HELOS-OASIS, SYMPATEC GmbH, Clausthal-Zellerfeld, Germany).

### 2.4. Structural Analysis

The internal structure of the particles can be visualized by cryogenic scanning electron microscopy (Cryo-SEM) [23]. Lir-MVLs of SPC 75:25 DEPC were diluted and dropped onto the conductive carbon binder. The sample stage was rapidly frozen in liquid nitrogen for 30 s, and then transferred to the preparation chamber under a vacuum. The pre-cooled samples were rapidly fractured with a cold knife and the internal structure was exposed after sublimation at −90 °C for 10 min to obtain the Cryo-SEM images.

The distribution of the lipid–aqueous phase within the formulation greatly affects the quality of MVLs, which can be visualized by laser confocal scanning microscopy (LCSM) [22,23,38]. Fluorescent labeled Lir-MVLs were prepared by marking the lipid phases and internal aqueous phases with Coumarin 6 and Cyanine 5 and scanned in Z-stack mode using an oil immersion lens (100×/1.4) of LCSM (LSM710, Carl Zeiss, Oberkochen, Germany).

It is possible that there is a correlation between the surface morphology of the MVLs and their drug release properties. The surface morphology of the Lir-MVLs with different SPC-DEPC ratios was determined by atomic force microscopy (AMF) (Cypher ES, Asylum Research, Santa Barbara, CA, USA). Lir-MVLs were evenly spread on freshly peeled mica sheets and scanned in tap mode (ACair) after a few moments of evaporation.

Micronuclear magnetic resonance (micro-NMR) (Acorn Area, Xigo nanotools, Bethlehem, PA, USA) was used to analyze the internal vesicle sizes of the Lir-MVLs with different SPC-DEPC ratios through T_2_ relaxation times.

### 2.5. Fluidity Analysis

To investigate the fluidity of lipid membranes of Lir-MVLs prepared using mixed phospholipids with different PTTs, differential scanning calorimetry (DSC) was used to study changes in membrane fluidity by measuring the thermal behavior of membranes [39,40]. Lir-MVLs with different SPC-DEPC ratios were subjected to DSC analysis (DSC1/200, Mettler Toledo, Greifensee, Switzerland) under a nitrogen flow from −40 to 80 °C with a heating rate of 5 °C/min to analyze the fluidity of the lipid membranes.

### 2.6. In Vitro Release

In vitro release studies of Lir-MVLs with different SPC-DEPC ratios were performed using a sample separation method [32]. Briefly, 0.1 mL of Lir-MVLs and 0.4 mL of phosphate-buffered saline were transferred to 2 mL micro-centrifuge tubes by placing the samples in a water bath oscillator with the temperature set at 37 °C and the rotation speed set at 100 rpm. Samples were collected at each time point and centrifuged at 1500 rpm for 5 min after the addition of 0.9 mL of saline. The precipitates were determined by HPLC and the cumulative release was calculated.

### 2.7. In Vivo Pharmacodynamics

db/db diabetes model mice (6–8 weeks, male, Beijing Zhishan Health Medical Research Institute Co., Ltd., Beijing, China) were randomly divided into 7 groups (n = 3), namely the positive control group (Lir-solution group), SPC, SPC 85:15 DEPC, SPC 75:25 DEPC, SPC 50:50 DEPC, SPC 25:75 DEPC group, and saline group. The mice were injected subcutaneously in the back of the neck with Lir-solution, a series of Lir-MVLs (7 mg·kg^−1^ Lir), and an equal volume of saline, respectively. At predetermined time intervals after administration, blood was collected from the tail tip of mice and the blood glucose concentration (BGC) was measured using a glucometer (ACCU-CHEK Guide Me, Roche, Basel, Switzerland).

Kunming mice (6–8 weeks old, male, Liaoning Changsheng Biotechnology Co., Ltd., Benxi, China) were randomly divided into three groups (n = 5) and injected subcutaneously at the back of the neck with Lir-MVLs of SPC 75:25 DEPC (7 mg·kg^−1^ Lir) and an equal volume of saline injection. The oral glucose tolerance test (OGTT) was administered at 1, 4, 7, and 10 days. Specifically, mice were fasted for 14 h, given 25% glucose at 2 g·kg^−1^ by gavage, and then the BGC was measured at different time points.

### 2.8. The Retention at the Local Injection Site

To evaluate the drug retention in the injection site, Cyanine 5 was selected to label the internal aqueous phase of Lir-MVLs, and a series of fluorescently labeled Lir-MVLs (Cy5-Lir-MVLs) with different SPC-DEPC ratios were prepared. Kunming mice (6–8 weeks old, male, Liaoning Changsheng biotechnology Co., Ltd., Benxi, China) were randomly divided into five groups (n = 3). The hair around the back of the mice was shaved, and Cy5-Lir-MVLs with different SPC-DEPC ratios were injected subcutaneously at the back of the neck. On days 0, 2, 4, 6, 8, 10, 12, 14, 16, 18, 20, 22, 24, and 26 after the administration, the mice were placed in a small animal in vivo imaging system (FX-PRO, Carestream, Rochester, NY, USA) to evaluate the fluorescence intensity at the injection site (λex = 590 nm, λem = 700 nm). The relative fluorescence intensity (RFI, %) was calculated according to Equation (2).

RFI (%) = Fluorescence intensity/Initial fluorescence × 100(2)

### 2.9. Pharmacokinetic

SD rats (6–8 weeks old, male, Liaoning Changsheng Biotechnology Co., Ltd., Benxi, China) were randomly divided into two groups (n = 5). Victoza^®^ and Lir-MVLs of SPC 75:25 DEPC were injected subcutaneously into the back of the rats’ necks (7 mg·kg^−1^ Lir). Blood samples were collected from the orbital venous plexus at 1, 2, 4, 8, 12, 24, 36, 48, 72, 96, 144, 192, and 240 h after administration, and centrifuged at 4000 rpm for 10 min. Then, 200 µL plasma samples were processed by solid-phase extraction using Sem as an internal standard. The concentration of Lir was measured using a liquid chromatography–mass spectrometer (API 4000TM, AB SCIEX, Foster City, CA, USA). Lir and Sem were separated on a Phenomenex C18 column (2.1 × 150 mm, 5 µm, 300 Å) at 40 °C using a mobile phase containing acetonitrile in 0.1% formic acid and deionized water in 0.1% formic acid. The concentration of acetonitrile increased linearly from 10 to 90% (*v*/*v*) in 5 min at a flow rate of 0.5 mL/min. Quantification was performed using multiple-reaction monitoring (MRM) of transitions between *m*/*z* 938.9 and *m*/*z* 1064.5 in the case of Lir, and transitions between *m*/*z* 1029.8 and *m*/*z* 136.3 in the case of Sem. The mass spectrometry operating parameters were as follows: gas temperature, 500 °C, capillary voltage, 4000 V, and nebulizer pressure, 30 psi. The DP, EP, CE, and CXP were 90 V, 11 V, 40 V, 12 V for Lir, and 120 V, 11 V, 88 V, and 12 V for Sem. Relative bioavailability was calculated according to Equation (3). T in the formula represents the test formulation (Lir-MVLs of SPC 75:25 DEPC), R represents the reference formulation (Victoza^®^), and D represents the dose administered.

F (%) = (AUC_T_ × D_R_)/(AUC_T_ × D_T_) × 100%) (3)

### 2.10. Statistical Analysis

All results were shown as mean ± standard deviation and n values were at least 3 unless otherwise stated. Statistical differences between groups were analyzed by one-way analysis of variance (ANOVA) and Tukey’s post hoc tests. * *p* < 0.05 was considered significant. ** *p* < 0.01 was considered highly significant.

## 3. Results and Discussion

### 3.1. The Basic Characterization of Lir-MVLs

After mixing, the Lir-MVLs appeared as a milky-white suspension, as shown in Figure S1. The morphology of Lir-MVLs was observed under an optical microscope, as shown in Figure S2, and it was seen that the Lir-MVLs were rounded and homogeneous in shape, without fragmentation or adhesion. The particles were internally dense with a non-concentric honeycomb structure. The EE and particle size of Lir-MVLs with different SPC-DEPC ratios are shown in Table 1, with the EEs all being above 90% and D_50_ all around 30 µm.

**Figure 2:**
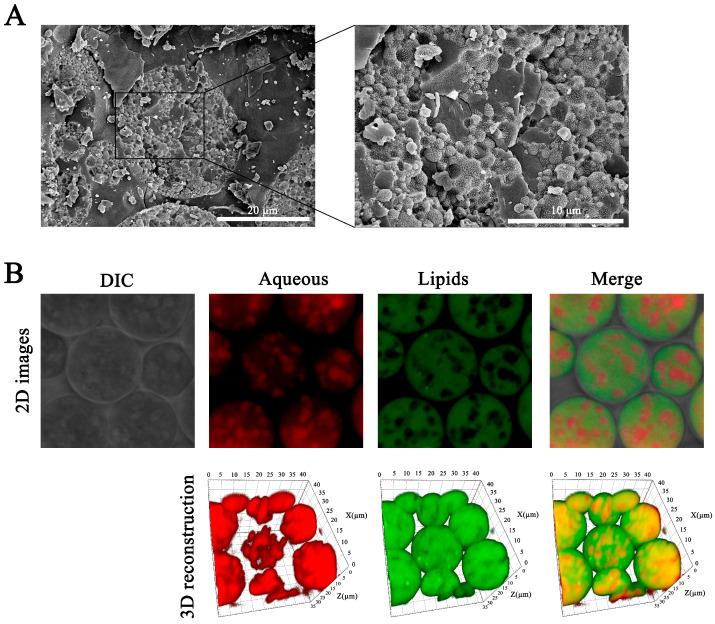
(**A**) The Cryo-SEM images of Lir-MVLs. (**B**) The distribution of the lipid–aqueous phase of Lir-MVLs.

As shown in Figure 2A, Lir-MVLs had a spherical morphology, and the cross-section revealed the typical non-concentric stacked vesicle-like structure of MVLs with a uniform distribution, and there were many micron-sized vesicles within the structure.

Figure 2B showed that the lipid phase of Lir-MVLs was continuously distributed and the internal aqueous phase had a vesicular structure, which was consistent with the results of Cryo-SEM. The detailed structure of MVLs can be seen in the DIC field of view, and the three-dimensional reconstruction showed the distribution of the lipid and aqueous phases of Lir-MVLs more intuitively, which was evidence of the structural formation of the MVLs.

### 3.2. The Fluidity of Lipid Membranes

**Figure 3:**
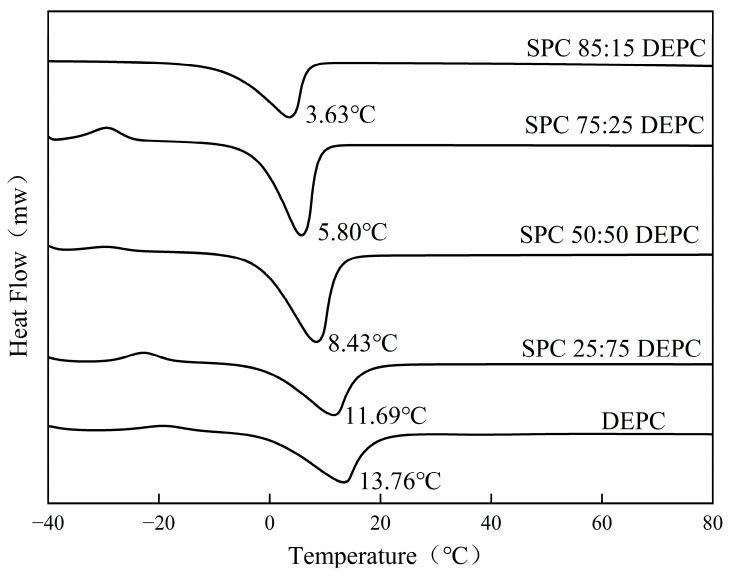
The DSC curves of Lir-MVLs.

The membrane fluidity of MVLs is critical in modulating drug release [41]. The DSC results of Lir-MVLs with different proportions of SPC-DEPC are shown in Figure 3 and Figure S3. As the temperature increases, a heat absorption peak appears on the DSC curve, representing the transformation of the lipid bilayer from the gel phase to the liquid crystal phase. The heat absorption peak of the DSC thermogram of Lir-MVLs of pure DEPC appears at 13.76 °C. As the proportion of SPC in the Lir-MVLs increased, the PTT decreased, indicating that the fluidity of the lipid membranes increased, which predicted faster drug release.

### 3.3. The Surface Morphology

**Figure 4:**
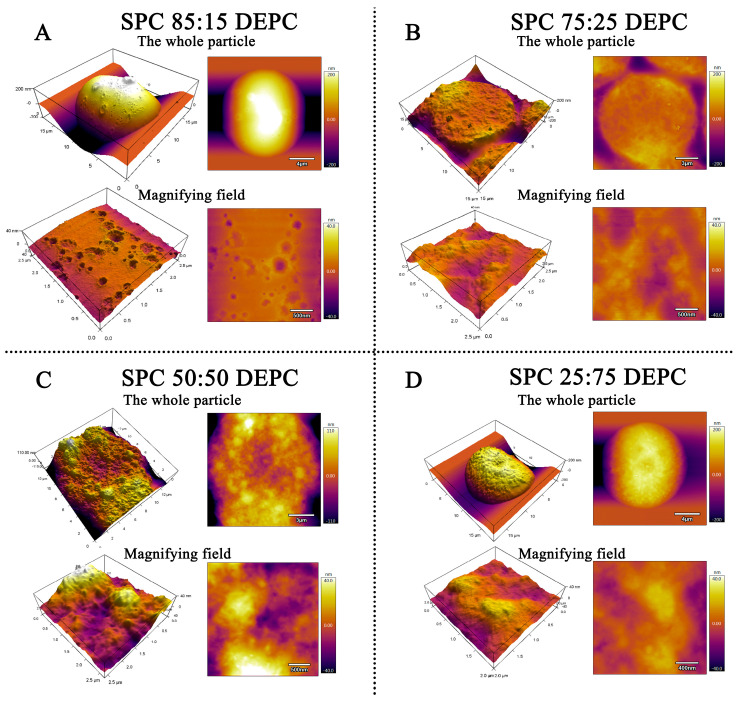
The AFM images of Lir-MVLs. (**A**) SPC 85:15 DEPC; (**B**) SPC 75:25 DEPC; (**C**) SPC 50:50 DEPC; and (**D**) SPC 25:75 DEPC.

The AFM images of Lir-MVLs with different SPC-DEPC ratios are shown in Figure 4. When the SPC-DEPC ratio was 85:15, the surfaces of the Lir-MVLs showed a large amount of crater-like damage, presumably caused by vesicle rupture, and the rest of the surface was smooth. This phenomenon was not observed on the surfaces of Lir-MVLs with SPC 75:25 DEPC, SPC 50:50 DEPC and SPC 25:75 DEPC, suggesting that the addition of SPC with lower PTT makes the surface of the Lir-MVLs more fragile and reduces their structural stability. It was also found that when the SPC-DEPC ratio was lower than 75:25, the proportion of SPC did not significantly change the surface morphology of the formulations. Therefore, it was speculated that 75:25 is the critical point of SPC-DEPC ratio, and the formulations with a proportion higher than this point would tend to release the drug more rapidly.

### 3.4. Characterization of Aqueous Phase–Particle Interfaces

As an emerging technology, micro-NMR has seen increasing use in recent years for the characterization of particle interfaces. Usually, a hydration layer is formed after the particles are dispersed in the dispersing medium, and the relaxation time of water protons in the hydration layer is lower than that of water molecules in the solution system. Therefore, this phenomenon can be used to investigate the properties of MVLs with W/O/W structures at the aqueous phase–particle interface [42]. For MVLs, the W/O/W structure results in two particle–water interfaces in the system, namely the outer water phase–particle interface and the inner water phase–particle interface. Relaxation time is essentially a response to the number of hydrated layers at the interface. For the same concentration, smaller particle sizes will typically have more hydrated layers, resulting in shorter relaxation times. The particle size of the MVLs is concentrated at 20–30 μm, and the particles settle overnight during the test so that they are closely stacked with each other, resulting in the formation of very few hydration layers at the particle interfaces in the external aqueous phase. The particle size variation in micron-sized MVLs has little effect on the relaxation time, but more on the volume and distribution of their internal chambers; that is, the number of hydration layers at the internal aqueous phase–particle interface.

**Figure 5:**
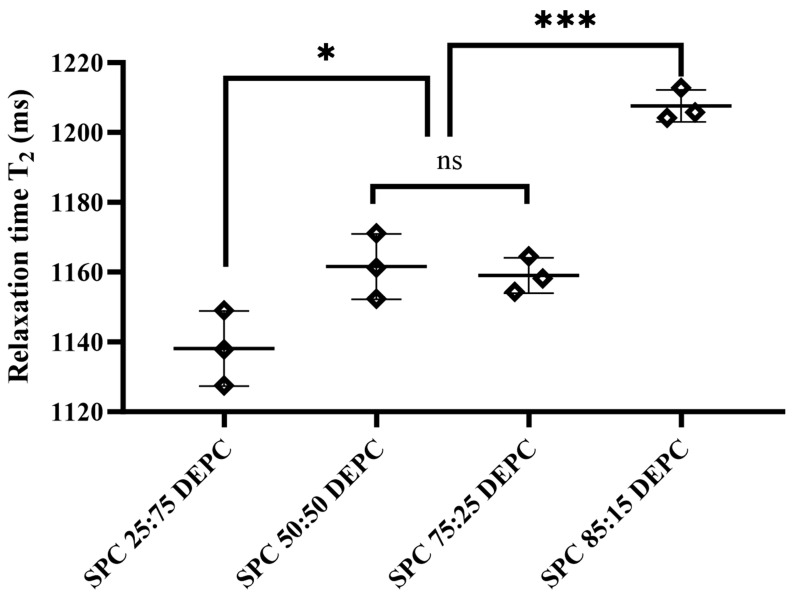
The relaxation time (T_2_) of Lir-MVLs. The significant differences between the two groups are expressed: * *p* ˂ 0.05, *** *p* ˂ 0.001, “ns” means no significant difference.

The results of the aqueous phase–particle interface characterization are shown in Figure 5 and Figure S4. The relaxation time T_2_ = 2168.2 ms for the external aqueous phase and T_2_ = 1100–1250 ms for the Lir-MVLs suggested that more particle–solvent interfaces were formed in the system. As shown in Figure 5, there was no significant change in the relaxation time as the SPC-DEPC ratio increased from 50:50 to 75:25, indicating that there was no significant change in the aqueous phase–particle interfaces within the SPC-DEPC at this ratio. As the SPC-DEPC ratio increased from 25:75 to 50:50 and from 75:25 to 85:15, the relaxation time T_2_ values of Lir-MVLs increased significantly, indicating that at this SPC-DEPC ratio, the number of internal aqueous phase–particle interfaces decreased with the increase in the SPC ratio in the MVLs, which resulted in the formation of fewer internal vesicles with larger sizes. Large vesicles were more prone to rupture and tended to release the drug more quickly than small vesicles [43].

### 3.5. In Vitro Release of Lir-MVLs Based on Mixed Phospholipids

**Figure 6:**
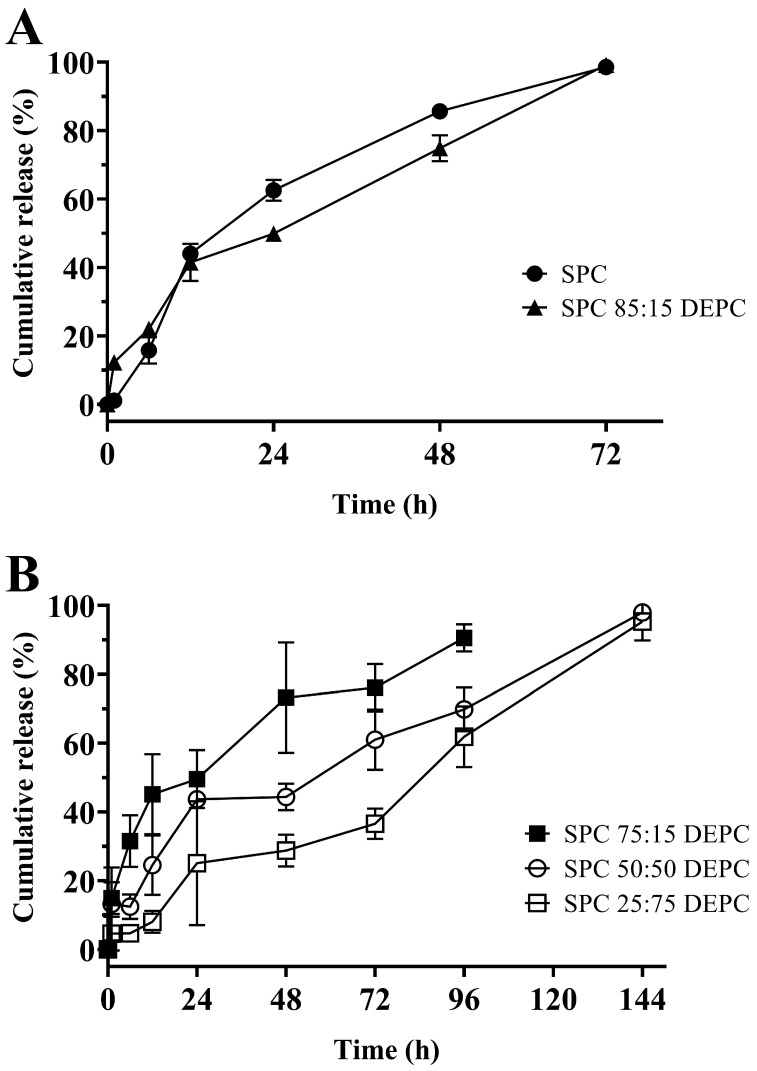
The cumulative release profiles of Lir-MVLs, (**A**) SPC, SPC 85:15 DEPC, (**B**) SPC 75:25 DEPC, SPC 50:50 DEPC, and SPC 25:75 DEPC.

The in vitro release behavior of MVLs is an important index for studying the drug release characteristics of sustained-release formulations, which can be used to understand the sustained-release duration, release rate, and drug release mechanism of the formulations. As can be seen in Figure 6, at 0–48 h, the formulation with an SPC-DEPC ratio higher than or equal to 75:25 released more than 70% of the total amount of drugs. The whole release process curve was smooth without the emergence of the platform period, indicating that the drug was released continuously. While the formulations with an SPC-DEPC ratio that was less than or equal to 50:50 had a better sustained-release effect (only 25–40% of the drug was released at 0–48 h), the release curve showed an obvious plateau at 24–48 h, during which the drug was essentially not released. The results showed that as the SPC ration increased, the release rate was accelerated, which was consistent with the lipid membrane features of the corresponding preparations. And when the SPC-DEPC ratio was less than or equal to 50:50, the phenomenon of delayed drug release occurred.

The in vitro release data were fitted to zero-order kinetics, first-order kinetics, and Higuchi and Korsmeyer–Peppas equations. Lir-MVLs of pure SPC were consistent with first-order release; SPC 85:15 DEPC, SPC 75:25 DEPC, and SPC 50:50 DEPC were consistent with the Korsmeyer–Peppas kinetic model; and SPC 25:75 DEPC was consistent with zero-level release. The fitting results are shown in Tables S1–S5. When the release data were consistent with the Korsmeyer–Peppas equation, the drug release mechanism in the Korsmeyer–Peppas equation predominantly exhibited diffusion for n ≤ 0.45, whereas for n ≥ 0.89 it predominantly showed signs of erosion, and in between, it included both diffusion and erosion. It could be seen that the release mechanisms of SPC 85:15 DEPC and SPC 50:50 DEPC were diffusion and erosion (n = 0.546, 0.545), whereas the main release mechanism of SPC 75:25 DEPC was diffusion (n = 0.370). The reason for this result was that when the SPC-DEPC ratio was 85:15, the lipid membrane was mobile, the structural stability was poorer, and the internal vesicles were larger and easily ruptured, and thus, the release mechanism tended to be a combination of diffusion and erosion. When the SPC-DEPC ratio was 75:25, the surface was more compact, the structure was more stable, and the lipid membrane fluidity was relatively favorable, resulting in a release mechanism that tended to be diffusion. When the SPC-DEPC ratio was 50:50, with the increase in the proportion of high PTT phospholipid DPEC, the structural stability improved, while the fluidity of the lipid membrane was further reduced, which caused the diffusion to be weakened, resulting in the drug release mechanism being more inclined toward a combination of diffusion and erosion. The above results suggested that different proportions of mixed phospholipids can alter the lipid membrane properties of MVLs, which can significantly modulate the drug release rate and release mechanism. As a complex formulation with multiple release units, after the rupture of the outer vesicles, the rest of the inner vesicles remain intact due to their special non-concentric honeycomb structure. Meanwhile, owing to the different diffusion distances between the outer and inner vesicles, the drug can show sequential diffusion release from the vesicles, which endows the MVLs with excellent sustained-release properties [32,33,44,45].

### 3.6. Hypoglycemic Effect in db/db Diabetes Model Mice

Different ratios of SPC-DEPC modulated the properties of the lipid membranes of Lir-MVLs, which in turn exhibited different release characteristics. Since the release behavior of the formulations may differ in vitro and in vivo, and in vivo experiments were further established to evaluate the hypoglycemic effect of Lir-MVLs with different SPC-DEPC ratios.

**Figure 7:**
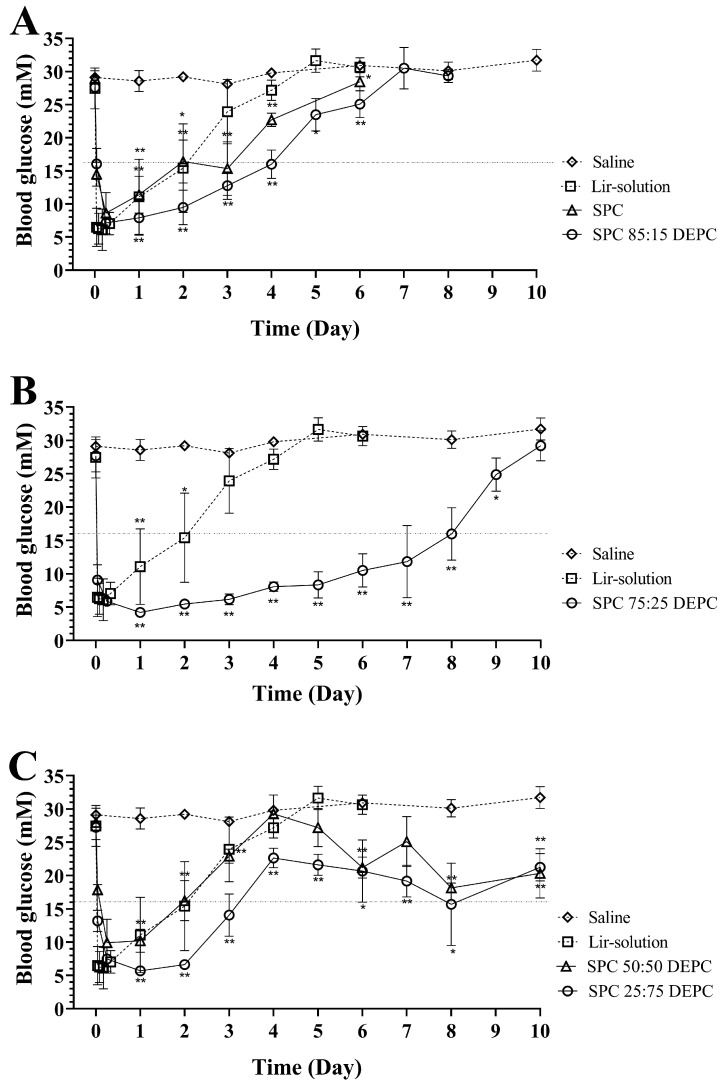
The mean BGC–time curve after subcutaneous drug delivery in db/db mice, saline, Lir-solution, and Lir-MVLs. (**A**) SPC, SPC 85:15 DEPC; (**B**) SPC 75:25 DEPC; (**C**) SPC 50:50 DEPC, SPC 25:75 DEPC; * *p* ˂ 0.05; ** *p* ˂ 0.01 vs. saline group.

The hypoglycemic experiment results in db/db diabetes model mice are shown in Figure 7. Lir-MVLs with different SPC-DEPC ratios showed different degrees of long-lasting hypoglycemic effects compared to the saline and Lir-solution groups. As can be seen in Figure 7A, after subcutaneous injection of Lir-solution, with Lir-MVLs of pure SPC and SPC 85:15 DEPC, compared with the control group, the BGC of db/db mice was rapidly reduced to below 10 mM and recovered to above 16 mM on day 3, day 4, and day 5, respectively. As can be seen in Figure 7B, after subcutaneous injection of Lir-MVLs of SPC 75:25 DEPC, the BGC of mice decreased rapidly to below 10 mM and recovered to above 16 mM on day 9. As can be seen in Figure 7C, after subcutaneous injection of Lir-MVLs of SPC 50:50 DEPC and SPC 25:75 DEPC, the BGC of mice could rapidly drop below 10 mM, but recovered rapidly above 16 mM on days 3 and 4, respectively. However, the BGC of these two groups showed a decreasing trend again after day 4 until day 10.

The above results indicate that when the SPC-DEPC ratio was more than and equal to 85:15, the sustained hypoglycemic effects were unsatisfactory due to their rapid drug release. While the SPC-DEPC ratio was less than 75:25, the BGC was unable to remain below 16 mM for more than 4 days, even though a certain hypoglycemic effect was exhibited for a prolonged period. Only Lir-MVLs of SPC 75:25 DEPC exhibited an effective long-lasting and stable hypoglycemic effect. The results of hypoglycemic experiments in db/db diabetes model mice have correlated well with in vitro release results. It is confirmed that by adjusting the mixed phospholipids proportions, the drug release could be more finely controlled to exert the expected therapeutic effect.

### 3.7. The Retention of Lir-MVLs at the Local Injection Site in Mice

**Figure 8:**
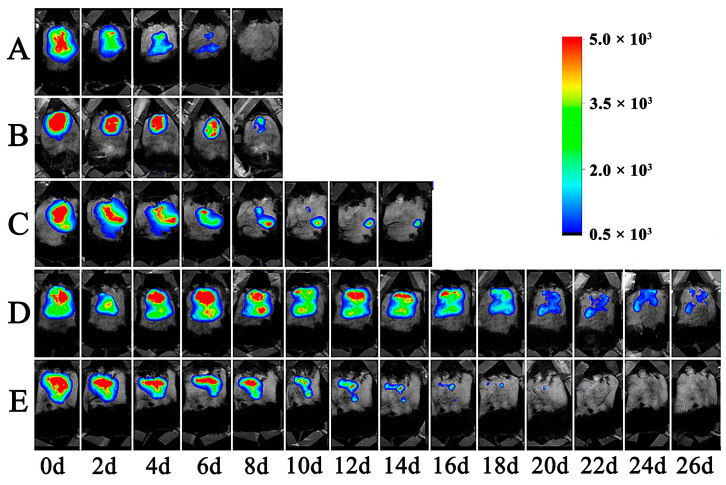
Fluorescence imaging results of Lir-MVLs at different time points at the injection site in mice. (**A**) SPC, (**B**) SPC 85:15 DEPC, (**C**) SPC 75:25 DEPC, (**D**) SPC 50:50 DEPC, and (**E**) SPC 25:75 DEPC.

**Figure 9:**
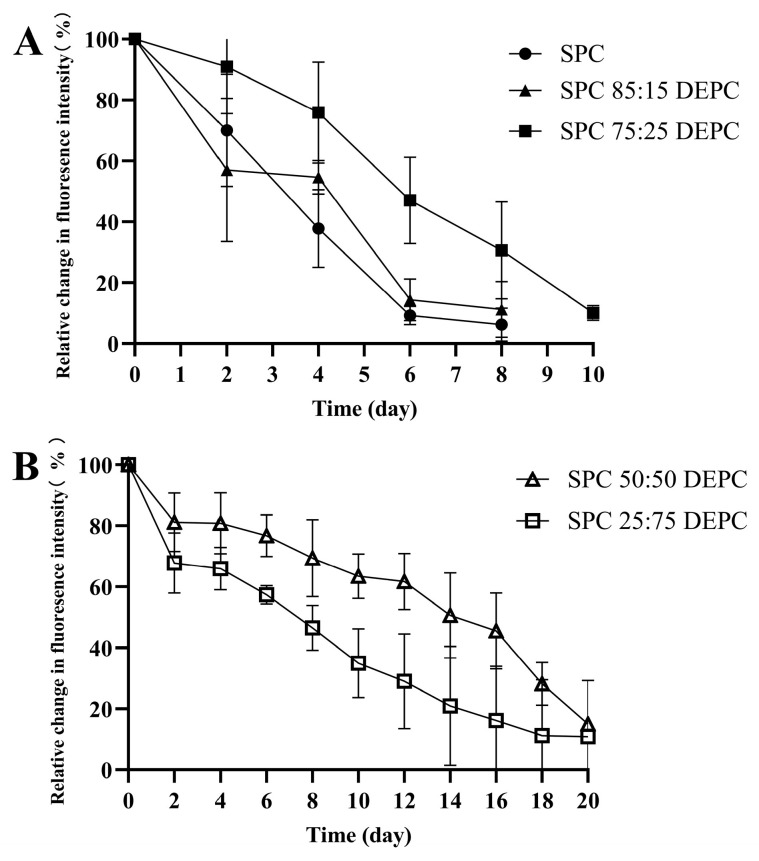
The relative change in fluorescence intensity–time curve after subcutaneous injection of CY5-Lir-MVLs in db/db mice. (**A**) SPC, SPC 85:15 DEPC, SPC 75:25 DEPC and (**B**) SPC 50:50 DEPC, SPC 25:75 DEPC.

As shown in Figure 8 and Figure 9, the retention times of Cy5-Lir-MVLs of SPC, SPC 85:15 DEPC, SPC 75:25 DEPC, SPC 50:50 DEPC, and SPC 25:75 DEPC in mice were 5 d, 6 d, 9 d, 20 d, and 16 d as the RFI decreased below 20%, respectively, and the retention time of SPC 50:50 DEPC was the longest. Local retention experiment results matched the in vitro release experiment and in vivo hypoglycemic experiment results. When the proportion of SPC-DEPC was greater than 75:25, the RFI decreased smoothly with time, suggesting that the release of Lir-MVLs was smooth at this time; however, the shorter retention led to a non-sustainable hypoglycemic effect. When the proportion of SPC-DEPC was less than 75:25, the RFI decreased by less than 5% on days 2–4 and the curve showed an obvious plateau, indicating an inadequate drug release during this period, which matched the transient increase in blood glucose in db/db mice on days 2–4. The longer retention time further confirms the slow release of the drug, resulting in an inadequate hypoglycemic effect. Only SPC 75:25 DEPC was at a suitable point. Considering these findings together, it is concluded that the mixed phospholipid ratio not only affected the local drug release rate of Lir-MVLs, but also had an obvious influence on the retention time at the local injection site, contributing to the sustained hypoglycemic effect.

### 3.8. Postprandial Blood Glucose Control in Healthy Mice

By exploring the characteristics of Lir-MVLs and combining the results of in vitro release and pharmacodynamics, Lir-MVLs of SPC 75:25 DEPC with good sustained-release properties were prepared, exhibiting a suitable release rate, retention time, and long-lasting hypoglycemic effect. To further validate the long-lasting and smooth postprandial blood glucose control effect of Lir-MVLs in healthy mice, and more accurately reflect the release process of the sustained and controlled release formulations in organisms, Lir-MVLs of SPC 75:25 DEPC was subsequently subjected to OGTT in healthy mice and in vivo pharmacokinetic study in rats.

**Figure 10:**
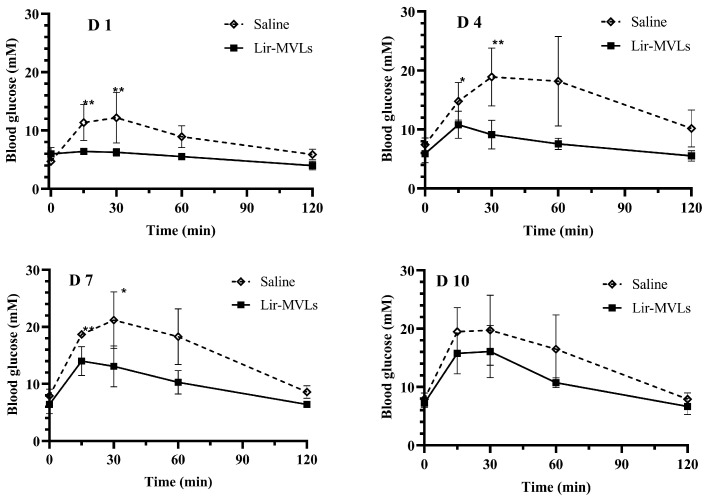
Blood glucose levels in each group after the OGTT were examined at a predetermined time (n = 5). The caption of “D” indicates the day after treatment. The significant differences between the two groups are expressed: * *p* ˂ 0.05, ** *p* ˂ 0.01.

OGTT can evaluate the ability of drugs to control postprandial blood glucose and improve the body’s glucose tolerance [46]. The results of the OGTT in healthy mice are shown in Figure 10. On days 1, 4, and 7, after 15 min of glucose administration, the BGC of mice in the control group increased to 11–18 mM, while that of mice in the Lir-MVLs group was controlled at 6–14 mM, which showed good control of BGC and did not fluctuate significantly. There was a significant difference in the results of the OGTT between the two groups on days 1, 4, and 7. After 120 min, the BGC of the mice in the Lir-MVLs group returned to basically normal levels, whereas those in the control group did not return to the initial BGC at 120 min on days 4 and 7, suggesting that repeated OGTT may damage the ability of mice to control blood glucose, while Lir-MVLs can effectively avoid this damage. Lir-MVLs of SPC 75:25 DEPC were able to control postprandial BGC at steady state on days 1,4, and 7, suggesting that the pharmacodynamic of Lir-MVLs of SPC 75:25 DEPC could be maintained for at least 7 days.

### 3.9. The Pharmacokinetic Profile in Rats

**Figure 11:**
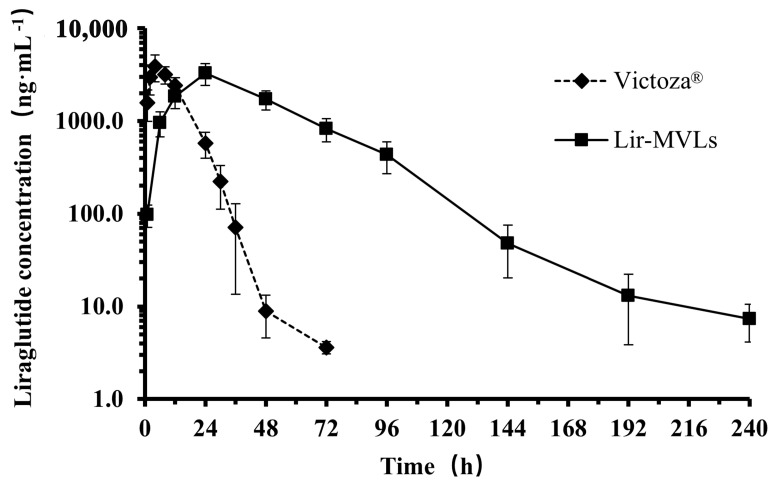
The mean plasma concentration–time curves of Lir in rats after subcutaneous injection of Victoza^®^ and Lir-MVLs. Data are shown as means ± SD, n = 5.

The plasma drug concentration–time curves of Lir following subcutaneous injection of Victoza^®^ and Lir-MVLs of SPC 75:25 DEPC in rats are shown in Figure 11. The plasma drug concentrations in the Lir-MVLs group were lower than those in Victoza^®^ group from 0 to 12 h and higher than those in Victoza^®^ group after 12 h. The concentration of Lir was below the lower limit of quantification at 72 h in Victoza^®^ group and 240 h in the Lir-MVLs group, respectively. The results indicated that subcutaneous administration of the Lir-MVLs of SPC 75:25 DEPC significantly prolonged the maintenance of blood concentration compared to the solution.

The plasma concentration data of the two groups were processed using the non-atrial model of the DAS 2.0 software. The results of the main pharmacokinetic parameters of the two groups are shown in Table 2. After subcutaneous injection of Lir-MVLs of SPC 75:25 DEPC, the maintenance time of plasma concentration was up to 240 h, which was significantly longer than that of Victoza^®^ (72 h). The mean retention time MRT_0–∞_ of Lir-MVLs group was 44.50 ± 4.56 h, which was 4.2 times longer than that of the Victoza^®^ group (*p* < 0.01). The time to peak was 24 h in the Lir-MVLs group, which was significantly longer than that of the Victoza^®^ group (4 h) (*p* < 0.01). The AUC_0–∞_ of the Lir-MVLs group was 2.82 times higher than that of Victoza^®^, and the relative bioavailability of the Lir-MVLs group was 282.3%. The results showed that Lir-MVLs of SPC 75:25 DEPC significantly prolonged the release time of the drug in vivo, decreased the peak blood concentration value, and prolonged the mean in vivo retention time of the drug.

According to the above results, it was speculated that MVLs had a large particle size of about 20–30 µm, much larger than 100 nm. After subcutaneous injection, they could escape from the capture of the endothelial reticular system, and were difficult for the capillaries to absorb, resulting in a slower metabolism rate. The encapsulation of Lir in MVLs protects Lir from degradation by DPP-IV or other non-specific enzymes in subcutaneous tissue, thereby improving relative bioavailability [29]. In addition, the drug was gradually released from MVLs by diffusion or erosion, which increased the retention time of the drug at the injection site and further delayed absorption. In a word, compared with the Victoza^®^ group, Lir-MVLs of SPC 75:25 DEPC in rats were able to significantly improve the pharmacokinetic behavior of Lir in rats, which also explained its long-acting hypoglycemic effect, which lasted for 8 days in db/db model mice.

## 4. Conclusions

This study showed that the MVLs based on biocompatible phospholipids could sustain the delivery of peptides, and the release rate of MVLs could be regulated by the ratio of phospholipids with different PPTs. The EE of Lir-MVLs with different SPC-DEPC ratios was above 90%, and the particle size was around 30 µm. As the proportion of SPC (PPT = −20 °C) increased, Lir-MVLs had more flexible lipid membranes, poorer structural stabilization, and fewer internal vesicles with larger particle sizes, leading to faster release and a shorter retention time. When the SPC-DEPC ratio was greater than 85:15, the drug release rate was too fast, so the sustained hypoglycemic effects were not effective. When the SPC-DEPC ratio was less than 50:50, the drug release rate was too slow to fully release the drug, resulting in an inability to achieve satisfactory hypoglycemic effects. Only when the SPC-DEPC ratio was 75:25 could the Lir-MVLs be released smoothly and slowly through a diffusion mechanism, which was able to maintain the BGC in db/db mice below 16 mM for 8 days, and control postprandial blood glucose in a steady state for 7 days with a relative bioavailability of 282.3%, indicating that the release properties of this ratio best met the pharmacological requirements of Lir. The present study achieved the goal of regulating the release characteristics by adjusting the types of phospholipids with different PTT in Lir-MVLs and obtained the expected sustained-release effect, which further meets practical clinical needs and provides a new theoretical basis and guidance for the design of injectable sustained-release peptide preparations.

## Figures and Tables

**Table 1:** The EE and particle size of Lir-MVLs.

Formulation	EE (%)	D_50_ (µm)
SPC	93.05 ± 0.02	28.93 ± 0.08
SPC 85:15 DEPC	93.04 ± 0.04	32.27 ± 0.16
SPC 75:25 DEPC	95.22 ± 0.05	31.15 ± 0.22
SPC 50:50 DEPC	98.74 ± 0.01	29.93 ± 0.23
SPC 25:75 DEPC	90.02 ± 0.04	32.74 ± 2.67
DEPC	93.10 ± 0.02	30.09 ± 0.53

**Table 2:** Pharmacokinetics parameters of Lir-MVLs of SPC 75:25 DEPC and Victoza^®^ after a single subcutaneous injection to rats with a dosage of 7 mg·kg^−1^.

	Lir-MVLs	Victoza^®^
AUC_0–t_ (mg·h·mL^−1^)	161 ± 32	57 ± 10 **
AUC_0–∞_ (mg·h·mL^−1^)	161 ± 32	57 ± 10 **
MRT_0–t_ (h)	44 ± 4	10.6 ± 1.3 **
MRT_0–∞_ (h)	44 ± 5	10.6 ± 1.4 **
T_max_ (h)	24 ± 0	4.0 ± 2.4 **
C_max_ (ng·mL^−1^)	3270 ± 860	3810 ± 1020

** *p* < 0.01.

## Data Availability

Data are contained within the article.

## Supplementary Materials

The following supporting information can be downloaded at https://www.mdpi.com/article/10.3390/pharmaceutics17020203/s1, Figure S1: The appearance of Lir-MVLs; Figure S2: Microphotograph of Lir-MVLs (magnification 10 × 40); Tables S1–S5: The results of the fitting equation and coefficients; Figure S3: The DSC curve of blank MVLs; Figure S4: The relaxation time (T_2_) curve of Lir-MVLs. (A) outer aqueous phase, (B) SPC 25:75 DEPC, and (C) SPC 50:50 DEPC, (D) SPC 75:25 DEPC, (E) SPC 85:15 DEPC.
