# Supplementary material for: Sustained Delivery of Liraglutide Using Multivesicular Liposome Based on Mixed Phospholipids
Source: Pharmaceutics. 2025 Feb 6;17(2):203. doi: 10.3390/pharmaceutics17020203 (PMC11859442; doi:10.3390/pharmaceutics17020203)
Supplement: Supplementary file 1 [file pharmaceutics-17-00203-s001.zip › pharmaceutics-3440736-supplementary.pdf]

pharmaceutics

Sustained delivery of liraglutide using multivesicular liposome based on mixed phospholipids

Runpeng Zhang, Xinyu Yao, Siqi Gao, Tingting Xu, Da Wang, Luping Sha, and Li Yang

1. Supplementary figures

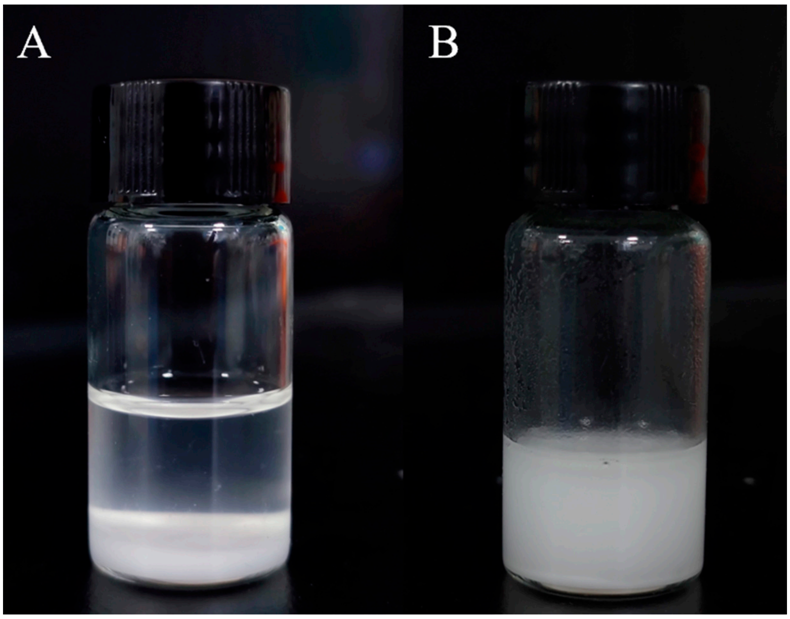

Figure S1. The appearance of Lir-MVLs (A) setting, (B) suspension

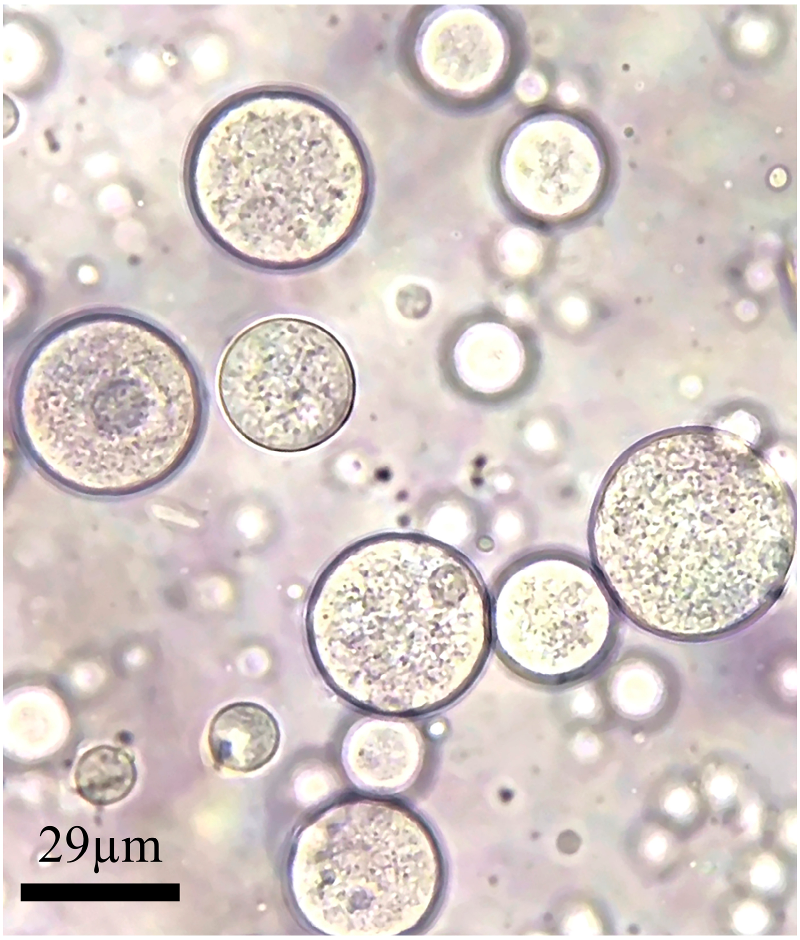

Figure S2. Microphotograph of Lir-MVLs (magnification 10×40)

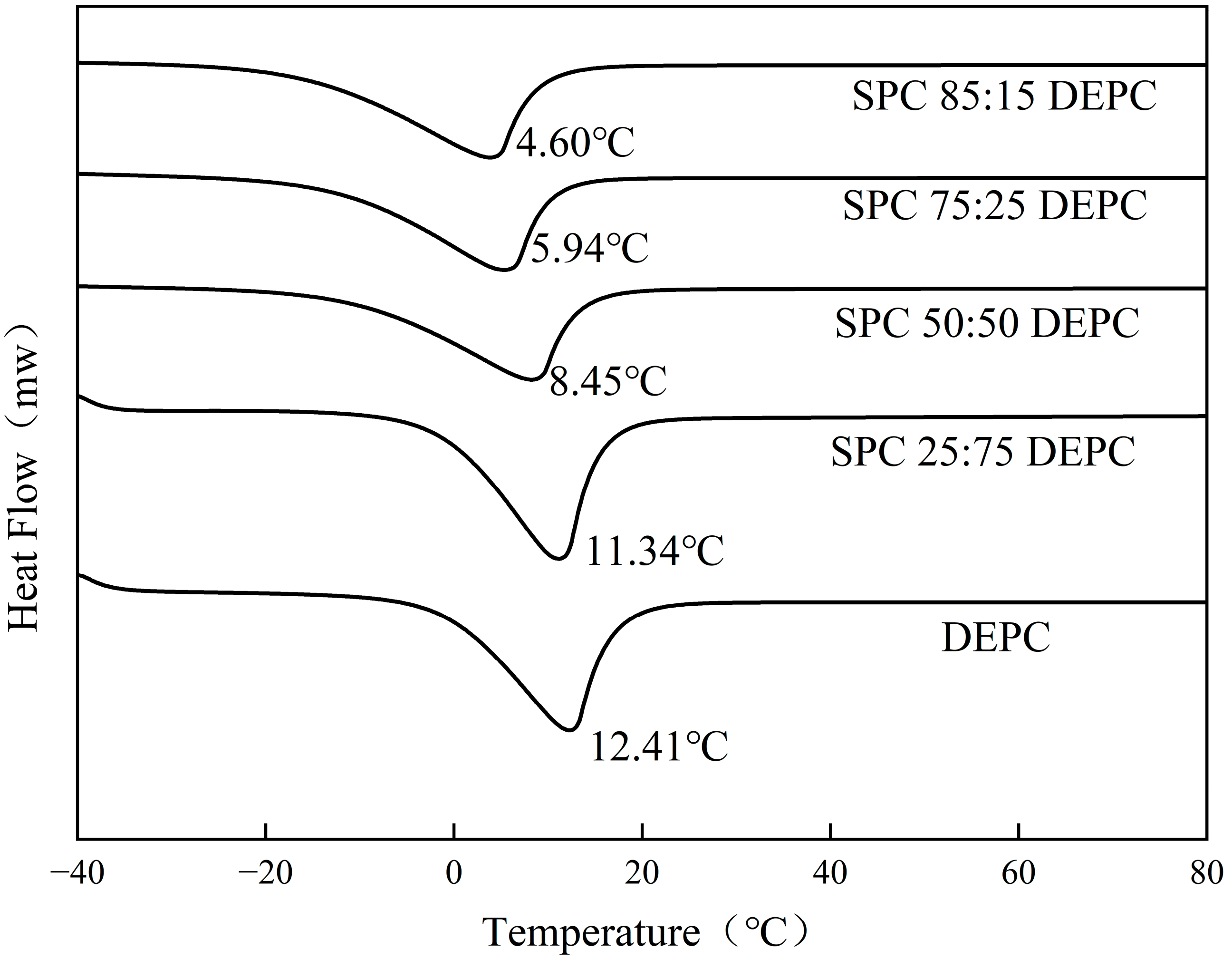

Figure S3. The DSC curves of Blank-MVLs

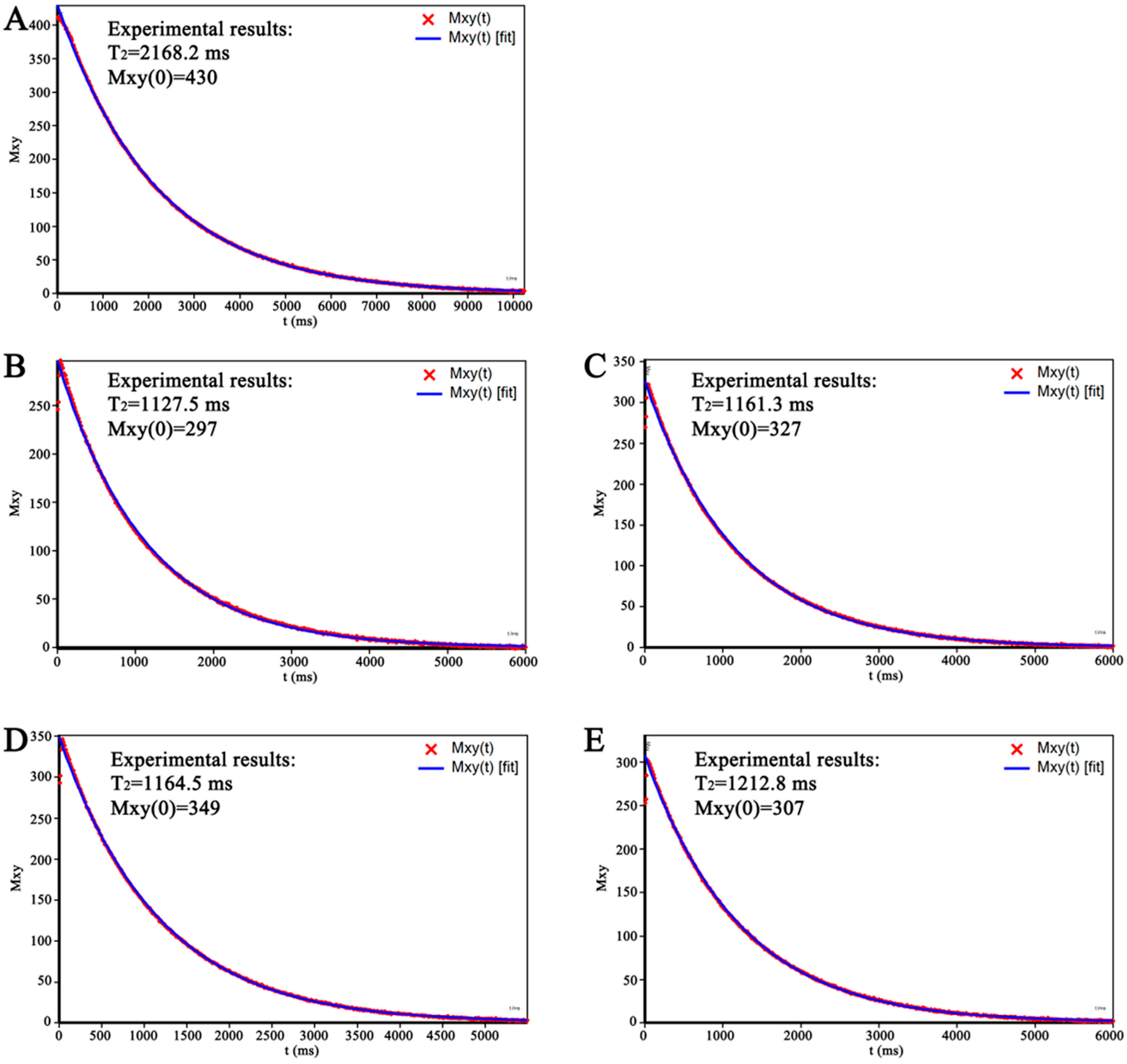

Figure S4. The relaxation time (T_2) curve of Lir-MVLs. A: outer aqueous phase, B: SPC 25:75 DEPC, C: SPC 50:50 DEPC, D: SPC 75:25 DEPC, E: SPC 85:15 DEPC.

2. Supplementary tables

Table S1. The results of fitting equation and coefficients of SPC(n=3)

Model	Equation	R^2
Zero-order	F=1.387t+11.645	0.8885
First-order	$F=100(1-e^{-0.042t})$	0.9911
Higuchi	$F=12.814t^{1/2}-5.907$	0.9738
Korsmeyer-Peppas	$F=9.2624t^{0.565}$	0.9706

Table S2. The results of fitting equation and coefficients of SPC 85:15 DPEC (n=3)

Model	Equation	R^2

Zero-order	F=1.258t+13.524	0.9439
First-order	$F=100(1-e^{-0.035t})$	0.9622
Higuchi	$F=11.363t^{1/2}-1.388$	0.9890
Korsmeyer-Peppas	$F=9.351t^{0.546}$	0.9911

Table S3. The results of fitting equation and coefficients of SPC 75:25 DPEC (n=3)

Model	Equation	R^2
Zero-order	F=0.803t+21.622	0.8641
First-order	$F=100(1-e^{-0.031t})$	0.8887
Higuchi	$F=8.761t^{1/2}+7.060$	0.9736
Korsmeyer-Peppas	$F=16.448t^{0.370}$	0.9901

Table S4. The results of fitting equation and coefficients of SPC 50:50 DPEC (n=3)

Model	Equation	R^2
Zero-order	F=0.612t+13.381	0.9387
First-order	$F=100(1-e^{-0.015t})$	0.9251
Higuchi	$F=7.561t^{1/2}-0.416$	0.9678
Korsmeyer-Peppas	$F=6.166t^{0.545}$	0.9702

Table S5. The results of fitting equation and coefficients of SPC 25:75 DPEC (n=3)

Model	Equation	R^2
Zero-order	F=0.623t+1.559	0.9745
First-order	$F=100(1-e^{-0.010t})$	0.9122
Higuchi	$F=7.231t^{1/2}-9.953$	0.8871
Korsmeyer-Peppas	$F=0.569t^{1.027}$	0.9694

Disclaimer/Publisher's Note: The statements, opinions and data contained in all publications are solely those of the individual author(s) and contributor(s) and not of MDPI and/or the editor(s). MDPI and/or the editor(s) disclaim responsibility for any injury to people or property resulting from any ideas, methods, instructions or products referred to in the content.
